# Disentangling the Correlates of Drug Use in a Clinic and Community Sample: A Regression Analysis of the Associations between Drug Use, Years-of-School, Impulsivity, IQ, Working Memory, and Psychiatric Symptoms

**DOI:** 10.3389/fpsyt.2014.00070

**Published:** 2014-06-24

**Authors:** Gene M. Heyman, Brian J. Dunn, Jason Mignone

**Affiliations:** ^1^Department of Psychology, Boston College, Chestnut Hill, MA, USA; ^2^Department of Psychology, Center for Studies in Behavioral Neurobiology, Concordia University, Montreal, QC, Canada; ^3^Prime, Buchholz & Associates, Inc., Portsmouth, NH, USA

**Keywords:** drug use, educational attainment, impulsivity, IQ, working memory, non-treatment drug users, methadone clinic, Cragg’s Double-Hurdle regression

## Abstract

Years-of-school is negatively correlated with illicit drug use. However, educational attainment is positively correlated with IQ and negatively correlated with impulsivity, two traits that are also correlated with drug use. Thus, the negative correlation between education and drug use may reflect the correlates of schooling, not schooling itself. To help disentangle these relations we obtained measures of working memory, simple memory, IQ, disposition (impulsivity and psychiatric status), years-of-school and frequency of illicit and licit drug use in methadone clinic and community drug users. We found strong zero-order correlations between all measures, including IQ, impulsivity, years-of-school, psychiatric symptoms, and drug use. However, multiple regression analyses revealed a different picture. The significant predictors of illicit drug use were gender, involvement in a methadone clinic, and years-of-school. That is, psychiatric symptoms, impulsivity, cognition, and IQ no longer predicted illicit drug use in the multiple regression analyses. Moreover, high risk subjects (low IQ and/or high impulsivity) who spent 14 or more years in school used stimulants and opiates less than did low risk subjects who had spent <14 years in school. Smoking and drinking had a different correlational structure. IQ and years-of-school predicted whether someone ever became a smoker, whereas impulsivity predicted the frequency of drinking bouts, but years-of-school did not. Many subjects reported no use of one or more drugs, resulting in a large number of “zeroes” in the data sets. Cragg’s Double-Hurdle regression method proved the best approach for dealing with this problem. To our knowledge, this is the first report to show that years-of-school predicts lower levels of illicit drug use after controlling for IQ and impulsivity. This paper also highlights the advantages of Double-Hurdle regression methods for analyzing the correlates of drug use in community samples.

## Introduction

According to journalists, public officials, and even popular song writers, kids who drop out of school early are inviting disaster. Not only are they undermining their future earnings, but life on the streets paves the way to delinquency, drugs, and worse. In his 1966 hit, “Don’t be a drop out,” rhythm and blues star James Brown warns that “without an education you might as well be dead,” and in an interview in the *New Yorker*, the current American Secretary of Education, Arne Duncan, comments that his friends who did not go to college are now casualties of drugs and crime (1). Yet, despite the widely assumed benefits of staying in school, the relationship between education and future prospects is not well understood. This is particularly true of drug use, the focus of the study described in this report. First, there is the question of the causal relations. There is evidence that early drug use causes students to drop out of school [e.g., Ref. (2, 3)], and, conversely, there is evidence that early success in school protects against later drug use (4, 5). Of course these are not mutually exclusive connections, and it is also possible that educational attainment and drug use are symptomatic of one or more common factors such as IQ and/or conscientiousness, so that school functions as a proxy for its correlates and is not itself a causal factor. For instance, educational attainment is correlated with IQ, psychiatric symptoms, and impulsivity (6–8), and each of these factors is also correlated with drug use in just the way predicted by the hypothesis that they are linked to drug use by way of years spent in school.

Our goal was to better understand the relations between drug use, cognition, impulsivity, and years-of-school. Our approach differed from previous studies in several ways. We recruited non-clinic drug users as well as clinic drug users. This increased the chances that our measures varied over a wide range, and it increased the likelihood that the sample reflected most drug users, since most drug users, including those who meet the criteria for dependence, do not make use of treatment facilities [e.g., Ref. (9)]. Second, we obtained cognitive, dispositional, and demographic correlates of drug use so that we could use multiple regression methods to test whether significant zero-order correlates remained significant when they were entered as one of several simultaneous predictors of drug use. For example, would impulsivity continue to predict drug use if differences in years-of-school, IQ, and psychiatric symptoms were controlled for? Third, we used a “two-tier” multiple regression method, an approach that is much more common in economics than in drug research. This method allowed us to distinguish between the predictors of ever using a particular drug and the predictors of how often the drug was used. This distinction is helpful when the subjects of a study do not all use the same drugs.

One of the convenient aspects of studying drug use in clinic populations is that all of the subjects use drugs and likely use many of the same ones. However, clinic populations may provide a distorted picture of drug use since most drug users do not take advantage of clinic services [e.g., Ref. (9–11)]. In principle, community samples provide the opportunity for more representative accounts of the determinants of drug use, but they come with their own challenges. For instance, most of our subjects did not use every drug that we were interested in evaluating. This resulted in data sets that included many zero frequencies, which is incompatible with the assumptions of ordinary least squares regression. Researchers interested in the determinants of consumer behavior face an analogous problem when one or more commodities of interest are purchased by some but not all consumers. Following their experience, we adopted Cragg’s Double-Hurdle regression method (1971). This is a two-tier approach, which allowed us to model the decision to use a drug and the frequency of drug use conditional on use as two separate stochastic processes. Probit regression analysis determined the predictors of ever using a drug, and Truncated Ordinary Least Squares regression determined the predictors of frequency of drug use.

The sort of complexity that applies to cognition and drug use applies to the relationship between impulsivity and drug use. The basic finding is that higher scores on measures of impulsivity are positively correlated with differences in drug use. This holds for studies in which impulsivity was measured by questionnaires [e.g., Ref. (12)], by choice procedures which used hypothetical rewards [e.g., Ref. (13)], and by choice procedures which pitted actual sooner smaller rewards against later larger rewards [e.g., Ref. (14, 15)]. However, impulsivity is correlated with IQ [e.g., Ref. (16)], working memory [e.g., Ref. (17)], and years-of-school [e.g., Ref. (18, 19)]. Possibly, then, just as years-of-school may be a proxy for differences in cognition, impulsivity may be a proxy for differences in cognition and/or years-of-school. Consequently, we proceeded in a two-step manner. First, we asked whether there was a correlation between impulsivity and drug use, as others have found, and then we asked whether the correlation held up when common correlates of drug use and impulsivity were included in the analysis. Thus, our study used multiple regression analyses to better understand the correlates of drug use, with emphasis on the distinction between years-of-school and cognition and, similarly, the possible distinctions between impulsivity, cognition, and years-of-school.

## Materials and Methods

### Participants

One hundred and eighty-four subjects participated in the study. Seventy-seven were recruited from Boston methadone clinics and the others were from Boston area communities. At the clinics, we distributed flyers that described the study and asked for paid volunteers. To recruit community subjects, we placed ads in neighborhood newspapers and online (boston.craigslist.org). The ads stated that paid volunteers were sought for a study on drug use. We excluded volunteers who reported a history of head injury or were younger than 21 or older than 65 years of age. To ensure that the subjects could read and understand the questionnaires, we asked for evidence of reading skill in English. We accepted anyone who was fluent in English and said they had graduated high school or passed a General Equivalency Test for high school. (How this criterion may have influenced the results is reviewed in the Section “Discussion.”) The clinic subjects were tested at their clinics; the community subjects were tested at the Behavioral Pharmacology Research Laboratory of McLean Hospital. All subjects signed an approved consent form and were informed that the study was designed so as to insure the subjects’ anonymity. The McLean Hospital Institutional Review Board for Human Subject Research evaluated and approved all procedures and the consent form.

### Materials and procedure

The study session consisted of a series of questionnaires and cognitive tests. The questionnaires obtained information on demographic characteristics, years-of-school (including technical training, nursing classes, hair styling classes, and so on), drug use history, impulsivity [Barratt Impulsiveness Scale (20)], and psychiatric symptoms [Symptom Check List-90-Revised (21)]. The cognitive procedures included short-term and working memory span tests (22) and the vocabulary and matrix reasoning subtests of the Wechsler Abbreviated Scale of Intelligence, WASI (23).

#### Short-term and working memory span tests

The verbal span tests asked subjects to recall a list of letters. The instructions and stimuli were displayed on a laptop computer. Each letter (78 point Lucinda Sands Unicode font) appeared for 1.5 s, one-after-the-other, with the series varying in length in pseudo-random fashion from three to eight letters. The end of the series was marked by a prompt showing three question marks. The subject was then asked to identify the just-displayed series of letters in the order that they had appeared on a prepared form. In the working memory version of this test, there was an additional “interference” task. Prior to each letter, the monitor displayed an array of colored circles and squares. The subject’s task was to count out loud the blue–green circles. At the completion of the count, the experimenter advanced the screen to the next letter in the span. Thus, in the working memory task, subjects were asked to keep in mind a series of letters while completing a counting task.

In the spatial cognition task, the screen displayed a side or diagonal of a rectangle, with an arrow head at one end to indicate directionality. Each line segment appeared for 1.5 s, and, as in the letter version of this task, the subject was asked to recall each display in the order that it appeared, writing down their responses on a prepared form. With one exception, the procedure was the same as the letter spans. Pilot tests indicated that this task was more difficult than the letter spans so that the longest spatial span was six rather than eight items.

Each of the four span tests was preceded by instructions and three practice spans to insure that the subject understood the task. The experimental events and stimuli were controlled by E-Prime software programs (Psychology Software Tools, Inc.).

#### Vocabulary and matrix reasoning subtests, Wechsler Abbreviated Scale of Intelligence

The vocabulary subtest consists of a list of 42 words that the subject is asked to define. Like its predecessors (the vocabulary subtests of the WISC-III and WAIS-III) the WASI verbal test is said to measure crystallized or acquired intelligence. The matrix reasoning subtest consists of a series of 35 different geometric and colored patterns that the subject completed by identifying the correct stimulus from a set of five choices. It is similar to the matrix reasoning subtest in the WAIS-III, and, like this test, is said to measure “non-verbal fluid reasoning and general intellectual ability.” On the basis of national norms, these two tests provide an estimate of full scale IQ. For instance, the correlation between the WASI and the WAIS-III IQ was 0.87 in a large national sample (23). Neither test was timed.

#### Measuring drug consumption

The subjects answered detailed questionnaires regarding their history of illicit drug use, alcohol consumption, and smoking. The questions were fashioned after those found in other drug research programs [e.g., *Addiction Severity Index* (24) and *Personal Drinking History Questionnaire* (25)]. The illicit drug questionnaire identified six drug categories: marijuana/hashish, hallucinogens, amphetamine, cocaine, opiates, and “other drugs.” For each drug the subjects were asked to describe their: (1) level of use, as measured in days per week, (2) mode of self-administration, and (3) periods of use at a given overall frequency, as measured in two different ways: calendar year and age. On the basis of these data, we estimated: (1) total occasions that a drug was used, (2) duration of “regular use,” where regular use was defined as three or more times a week for a year or more, and (3) the current pattern of use. The questionnaires for smoking and drinking followed a similar format, yielding similar measures.

#### Urine sampling for current drug use

Methadone clinic subjects provided urine samples so that we could test for current drug use. The assay (Instant-View Multi-Drug Screen Urine Test) checked for the presence of 12 drugs, including morphine, methadone, various stimulants, benzodiazepines, marijuana/hashish, MDMA, and PCP.

#### Impulsiveness and current psychiatric symptoms

The Barratt Impulsiveness Scale (20) is a widely used, 30-item self-report questionnaire. Subjects rate themselves on a four point scale on questions regarding planning, spontaneity, patience, and susceptibility to boredom.

The Symptom Check List-90-R (SCL) is a self-report, psychiatric symptom inventory. It asks subjects to rate the degree to which they experienced 90 different symptoms as stressful over the previous 7 days. On the basis of research and clinical experience, the 90 verbal descriptions are grouped into nine symptom categories, plus a residual category. The categories are generally accepted symptom clusters, such as depression and anxiety. The SCL rating scale has five levels, ranging from “not at all” stressful to “extremely” stressful.

#### Demographic questionnaire

We also administered a questionnaire that gathered demographic information, including age, marital status, ethnicity, and years spent in school. School was broadly defined as any type of training, including hair dressing, nursing, and other forms of preparation for occupational roles.

#### Statistical analyses

We used multiple regression methods to identify the variables that predicted differences in drug use. This approach entailed two challenges. First, the drug frequency distributions were positively skewed, and, second, many subjects did not use a particular drug so that there were many “zero” scores in the frequency distributions. To correct for positive skew we transformed the frequencies so that they would better approximate a normal distribution. Guided by Stata’s Box-Cox test, we evaluated various “power” transformations of the frequencies, ranging from logarithmic to 0.5 (square root) and chose the two that most closely produced a normal distribution in drug use frequencies according to the Shapiro–Wilk test and visual inspection of the probability graphs (see Results).

The list of methods for analyzing data sets with a large number of zeroes includes logistic models, zero-inflated Poisson regression, and “two-tier” Tobit and Double-Hurdle regression analyses. Econometric researchers developed the two two-tier methods as a way of quantifying the likelihood of purchasing a good (participation) and then conditional on the purchase, the frequency of purchases (26, 27). Although both methods are widely used in economics, the Double-Hurdle has the advantage of allowing the researcher to evaluate whether the predictors of participation and predictors of frequency of participation differ. For instance, when the two sets are the same, the Double-Hurdle reduces to the Tobit analysis. Thus, Cragg’s Double-Hurdle approach is more flexible and inclusive, so we used it [see Ref. (28) for a helpful introduction to the method]. The first tier uses a probit regression equation to model whether an individual ever used a particular drug or not. The second tier uses a truncated regression equation to model the frequency of drug use, conditional on having used the drug at least once. Thus, each tier yields a set of regression coefficients and their significance levels. We entered the same set of predictors for both tiers, but this was not necessary. The analyses were conducted with SYSTAT 13 and Stata 12 statistical software.

## Results

### Demographics

The average age of the subjects was 40.7 (10.04) years, 57% were female, 73% were White, and the average number of years-of-school was 14.3 (2.6) – parentheses enclose the standard deviations. With one exception, the community and clinic demographic characteristics were quite similar. The average ages differed by <2 years (40.0 and 41.7), the proportions of males and females were nearly the same (58 and 56%), and the proportions of whites and non-whites were also similar (31 and 25%), respectively. However, community subjects typically spent more time in school than did clinic subjects. Almost all community subjects graduated high school and the majority had some post high school training (73%). In contrast, 34% of the clinic subjects earned a GED degree rather than graduating high school, and 60% went no further than the 12th grade. Overall, the average difference in years-of-school for the two groups was 3.5, which was statistically significant [12.3 and 15.8 years; *F*(1,182) = 135.4, *p* < 0.0005].

### Number of drug use occasions

Table 1 summarizes the reported levels of illicit and licit drug use for all subjects and for clinic and community subjects taken separately. For a given drug, consumption levels varied widely, and for every drug the average consumption level was considerably greater than the median consumption level, implying that the frequency distributions were positively skewed (as pointed out in the Section “Materials and Methods”). The community and clinic drug frequency distributions were also positively skewed, with the exception of cigarette smoking in clinic subjects. Also note that with the exception of stimulants, the maximum consumption level for a given drug was about the same for community and clinic subjects. Indeed, the maximum consumption level for a given drug was as likely to come from the community sample as the clinic sample. However, there were also differences in drug use. On average, drug consumption was much higher in the clinic sample, particularly for illicit drugs, and, in line with this finding, more community subjects reported little or no use of a particular drug. Consequently, the median consumption levels for community subjects were often zero. Put somewhat differently, the range of drug use frequencies was wider in the community sample, with many community subjects reporting no drug use and others reporting levels that matched the clinic subjects.

**Table 1:** **Frequency of drug use**.

	All subjects				Community				Clinic			
	Min.	Med.	Avg.	Max.	Min.	Med.	Avg.	Max.	Min.	Med.	Avg.	Max.
Opiates^a^	0	0	1468	13,416	0	0	204	13,416	130	2548	3225	11, 284
Stimulants^b^	0	12	876	12,064	0	12	225	5102	0	1300	1780	12, 064
Marijuana^c^	0	221	1464	11,284	0	221	750	9282	0	1456	2456	11, 284
Years regular^d^	0	4.5	8.5	34	0	4.5	2.6	34	3	15	16.7	34
Total occasions^e^	0	1508	4375	25,351	0	1508	1281	25,351	874	7332	8702	24, 128
Alcohol binges^f^	0	48	728	9072	0	48	452	8640	0	432	1112	9072
Cigarettes/100^g^	0	538	1066	6388	0	538	561	6388	0	1752	1768	5183

*^a^*F*(1,182) = 1034, *p* < 0.001; ^b^*F*(1,182) = 177, *p* < 0.001; ^c^*F*(1,182) = 57, *p* < 0.001; ^d^*F*(1,182) = 164, *p* < 0.001; ^e^*F*(1,182) = 195, *p* < 0.001; ^f^*F*(1,182) = 21, *p* < 0.001; ^g^*F*(1,182) = 66, *p* < 0.001*.

**Figure 1:**
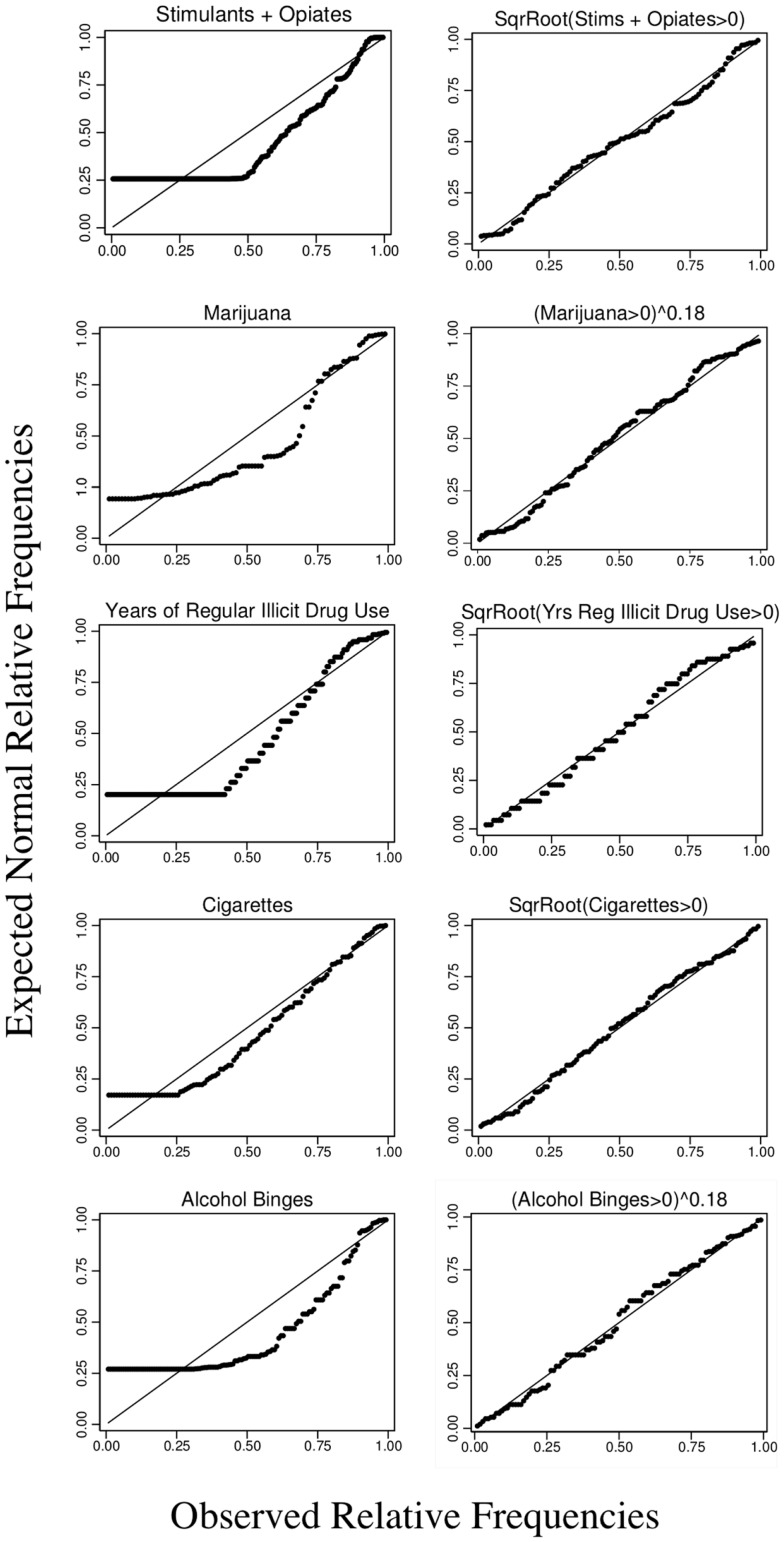
**Normal probability graphs of frequency of drug use and years of regular illicit drug use (>2/week for a year or more)**. The diagonal line plots a perfect correlation between the observed relative frequencies of use and the predicted relative frequencies assuming a normal distribution. The left panels show the untransformed frequencies including subjects who reported no use. The right panels show the transformed frequencies (square root or power with an exponent of 0.18) for subjects who used one or more times.

Figure 1 shows normal probability plots for our five measures of drug use. We combined the frequencies of opiate and stimulant use into a single category because they were highly correlated with each other (*r* = 0.77) and doing so resulted in a more orderly distribution of drug use frequencies, while maintaining a faithful reflection of each drug. (The correlation between the frequency of opiate use and the combined score was *r* = 0.92, and the correlation between the frequency of stimulant use and the combined score was *r* = 0.91.) On the *x*-axes are the obtained frequencies of drug use, and on the y-axes are the expected frequencies assuming that they were normally distributed. Thus, deviations from a straight line fit indicate deviations from normality. The left panels indicate that the untransformed frequencies were not normally distributed. The right panels show the same data transformed as indicated in the graph. We used Stata’s Box-Cox test to guide the search for the transformation that provided the best fit to a normal distribution, and on the basis of these results and visual inspection of the graphs, we settled on the square root transformation for stimulants and opiates, years of use and cigarettes, and power transformations of 0.18 for alcohol binges and marijuana use. According to the Shapiro–Francia test, the transformed drug use frequencies did not deviate significantly from normality, with the exception of marijuana (*p* < 0.05). However, as indicated by the graph, the distribution of marijuana use frequencies was not that different from the frequency distributions for other drugs. Thus, the transformed drug use frequencies approximated the regression model ideal of normally distributed variables. (Normality tests of the residuals are presented with the regression results.)

### Cognitive, psychiatric, and drug use correlations

Table 2 lists the correlations between the number of times a drug was used, the six cognitive measures, impulsiveness, the total scores on the psychiatric symptoms check list, and years-of-school. The asterisks indicate significance at the 0.05 level, taking into account the number of comparisons (Bonferroni corrected).

**Table 2:** Drug use, cognition, psychological disposition, and years-of-school correlations*.

	Letter STM^+^	Letter WM^+^	Spatial	Spatial	IQ vocab	IQ Matrix	Impulsiveness^+^	Psych sympt	Years
			STM	WM	test	teas test		total^+^	school
Opiate	−0.26*	−0.39*	−0.24*	−0.31*	−0.53*	−0.24*	0.37*	0.35*	−0.61*
Stimulant	−0.32*	−0.37*	−0.17*	−0.25*	−0.48*	−0.28*	0.31*	0.28*	−0.56*
Stimulant and opiate	−0.31*	−0.42*	−0.24*	−0.31*	−0.57*	−0.27*	0.39*	0.37*	−0.66*
Marijuana	−0.11	−0.14	−0.08	−0.22*	−0.35*	−0.21*	0.30*	0.33*	−0.46*
Years regular	−0.26*	−0.39*	−0.20*	−0.32*	−0.58*	−0.34*	0.38*	0.39*	−0.66*
Alcohol	−0.02	−0.04	−0.05	−0.15*	−0.26*	−0.21*	0.32*	0.25*	−0.36*
Cigs	−0.24*	−0.31*	−0.17*	−0.30*	−0.41*	−0.32*	0.25*	0.24*	−0.53*
Years school	0.31*	0.39*	0.21*	0.37*	0.63*	0.39*	−0.41*	−0.39*	

*p < 0.05, Bonferroni corrected.

*^+^STM refers to short-term memory; WM refers to working memory; Impulsiveness refers to the participant’s score on the Barratt questionnaire; Psych sympt total refers to the participant’s total score on the Symptom Check List-90 questionnaire. Correlations are based on the transformed drug frequencies*.

Most of the correlations were statistically significant, and the pattern of correlations revealed an underlying order across drugs and psychological measures. The correlations were highest for opiates and stimulants and lowest for alcohol. Second, although the absolute magnitudes of the correlations varied as a function of drug, the relative strengths of the correlations did not. For every drug, the correlations between frequency of use and working memory were stronger than the correlation between frequency of use and simple memory. For every drug, the correlations between frequency of use and verbal (letter) working memory span tests were stronger than the correlation between frequency of use and spatial (line) working memory span tests. And, similarly, for every drug the correlations with frequency of use were higher for the vocabulary component of the IQ test than for the matrix reasoning component of the IQ test. That is, for every drug, verbal cognition was a stronger correlate of frequency of use than was spatial cognition. There was an analogous orderliness to the dispositional measures (psychiatric symptoms and impulsivity). In six of the eight tests, impulsivity was more strongly correlated with drug use than were psychiatric symptoms. However, these differences were quite small.

The last column of Table 2 reveals that the strongest correlate of drug use was years-of-school. This was true for every drug, with the correlations varying from −0.36 (alcohol) to −0.66 (stimulants/opiates and years of illicit drug use). Also notice that years-of-school was significantly correlated with the cognitive measures and with impulsiveness and psychiatric symptoms. These correlations mirror the drug correlations in that they were stronger for the verbal tests, stronger for the working memory tests, and stronger for impulsiveness than for psychiatric symptoms. Thus, years-of-school was most strongly correlated with those variables that were most strongly correlated with drug use, and the rank order of the correlations was nearly identical for every drug, with scores on verbal tests outpacing scores on spatial tests.

### Current drug use

We also evaluated the relations between current drug use and cognition. Twenty-nine of 77 clinic subjects tested positive for one or more illicit drugs. However, their scores on the cognitive tests were similar to those of the clinic subjects who did not test positive for an illicit drug. For instance, the average IQ scores for the two groups were nearly identical: 94.6 and 94.0, none of the differences were statistically significant, and on two of the six tests, those who tested positive for drug use scored slightly higher. On the basis of verbal reports, 34 clinic subjects reported that they had used one or more illicit drugs in the 30 days prior to the interview. That is, verbal reports provided somewhat higher rate of recent drug use than did metabolic tests, a result reported by others as well [e.g., Ref. (29)]. The cognitive scores for those who reported past month drug use were similar to the scores of those who reported no drug use in the last 30 days, and none of the differences were statistically significant. We also asked community subjects about current drug use. Twenty-nine met the criteria for lifetime regular drug use, and of this subset, six reported that they had used an illicit drug at least once in the last 30 days. This is a very small sample. Nevertheless, we should report that their cognitive scores were about the same as those of subjects reporting no recent drug use (e.g., average IQ scores of 109.3 and 106.6, respectively).Thus, there was no evidence that current drug use influenced performance on the cognitive tests.

### Multiple regression analyses of the correlates of drug use

Years-of-school, scores on the cognitive tests, and scores on the dispositional questionnaires were correlated with one another as well as with drug use. To better understand the structure of the correlations we recalculated them using multiple regression methods. First, we applied principal component analyses to reduce the number of predictors and to establish predictors that were not correlated with each other (with varimax rotation of the factors). Three factors accounted for 81% of the variance in the six cognitive tests. These will be referred to as “IQ,” “verbal cognition,” and “spatial cognition,” with the labels identifying the cognitive test or tests that the factor was most strongly correlated with. Two factors accounted for 80% of the variance in Barratt scale results and the 10 Symptom Check-List symptoms. These will be referred to as “impulsiveness” and “psychiatric symptoms,” in accordance with their correlates. Thus, we reduced 17 cognitive and dispositional predictors of drug use to 5, and in each set the predictors were statistically independent of one another. To these five predictors we added three demographic predictors of drug use: age, gender, and “clinic status.” The latter was a binary measure that distinguished clinic and community subjects. Our goal was to dissociate being in treatment and years-of-school. For instance, if years-of-school was largely a proxy for clinic status then including clinic status in the analysis would reduce the correlation between years-of-school and drug use, perhaps to the extent that it was no longer a significant correlate of drug use. However, since a prerequisite for treatment was a diagnosis of opiate dependence, clinic status was necessarily a significant predictor of illicit drug use, and Table 1 suggests it may also predict drinking bouts and cigarette smoking. Thus, it was not obvious that the correlates of drug use listed in Table 2 would remain significant when clinic status was included in the regression equations.

### Regression (cragg’s double-hurdle) analyses of the correlates of drug use

Tables 3 and 4 summarize the regression analyses for licit and illicit drugs. For each drug – identified in the top row – the first two columns list the coefficients for the predictors of any use and their significance levels, as determined by probit regression. The third and fourth columns list the coefficients of the predictors and their statistical significance for the frequency of use among users (truncated regression), that is, conditional on use. The significance levels were set according to Stata’s “robust” command, which corrects for heteroskedastic residuals and observations that might have endue influence (“leverage”). We eliminated spatial cognition and verbal cognition from the regression analyses because preliminary tests revealed that their fitted coefficients were not significantly different from zero for any drug. Stata’s multicollinearity test among the predictors resulted in a median Variance Inflated Factor of 1.37 (or, alternatively, median tolerance of 0.75), which is well below the recommended maximum of 4.0 or 5.0. According to the Shapiro–Wilk test, the residuals were normally distributed, with the exception of those for smoking. For both licit and illicit drugs the Double-Hurdle regression models were significant [with an average Wald χ^2^ (7) = 459.9, *p* > 0.0000].

**Table 3:** **Double-Hurdle (Cragg’s) regression analysis: coefficients, robust standard errors, and significance**.

	Stimulants/opiates				Marijuana				Years regular use			
Predictors	Use/probit	*p*	Freq|Use TruncReg	*p*	Use/probit	*p*	Freq|Use TruncReg	*p*	Use/probit	*p*	Freq|Use TruncReg	*p*
Age	0.020 (0.014)	0.164	1.05*** (0.247)	0.000	−0.004 (0.010)	0.705	0.021*** (0.009)	0.017	0.001 (0.014)	0.960	0.057*** (0.009)	0.000
Gender	0.344 (0.307)	0.264	10.61*** (5.26)	0.001	0.177 (0.216)	0.414	0.562*** (0.171)	0.001	0.556 (0.293)	0.058	0.787*** (0.170)	0.000
Clinic	5.99*** (0.247)	0.000	39.19*** (9.36)	0.000	0.639* (0.275)	0.020	0.485 (0.248)	0.051	5.97*** (0.251)	0.000	0.934*** (0.251)	0.000
IQ	−0.012 (0.015)	0.409	0.264 (0.227)	0.246	0.007 (0.010)	0.506	−0.004 (0.009)	0.616	−0.122 (0.015)	0.401	−0.003 (0.007)	0.683
Impulsivity	0.576** (0.194)	0.003	0.771 (3.112)	0.805	0.168 (0.113)	0.138	0.113 (0.104)	0.278	0.333 (0.176)	0.063	−0.0370.100	0.711
Psych symptoms	0.248 (0.205)	0.226	−0.234 (2.547)	0.927	0.168 (0.113)	0.529	0.179 (0.093)	0.055	0.236 (0.189)	0.212	0.054 (0.093)	0.561
Years school	−0.145* (0.071)	0.038	−5.53*** (1.73)	0.001	−0.097 (0.054)	0.076	−0.055 (0.060)	0.364	−0.139 (0.072)	0.052	−0.139*** (0.059)	0.019

**Table 4:** **Double-Hurdle (Cragg’s) regression analysis: coefficients, robust standard errors, and significance**.

	Cigarettes				Alcohol bouts			
Predictors	Use/probit	*p*	Freq|Use TruncReg	*p*	Use/probit	*p*	Freq|Use TruncReg	*p*
Age	0.035** (0.011)	0.002	8.71*** (1.13)	0.000	−0.029** (0.010)	0.005	0.019 (0.010)	0.058
Gender	−0.100 (0.230)	0.662	12.76 (27.86)	0.647	0.913*** (0.224)	0.000	0.451** (0.168)	0.007
Clinic	0.819** (0.297)	0.006	61.22 (37.59)	0.103	0.422 (0.300)	0.154	0.095 (0.234)	0.685
IQ	−0.017 (0.011)	0.122	−1.04 (1.06)	0.324	0.0.003 (0.010)	0.726	−0.008 (0.006)	0.210
Impulsivity	0.056 (0.142)	0.691	6.91 (14.00)	0.622	0.254* (0.113)	0.025	0.282** (0.092)	0.002
Psych symptoms	−0.151 (0.134)	0.259	12.68 (10.14)	0.211	0.089 (0.111)	0.425	0.052 (0.083)	0.531
Years school	−0.154* (0.061)	0.012	−8.84 (7.83)	0.259	−0.052 (0.057)	0.369	−0.031 (0.046)	0.511

### Illicit drugs

Table 3 shows that the statistically significant predictors of any use and frequency of use conditional on any use differed. Clinic status was, of course, a significant predictor of any illicit drug use. Impulsivity was a significant predictor of any stimulant and opiate use, and years-of-school was also a significant predictor of any opiate and stimulant use and had a null-hypothesis *p* value of 0.052 for one or more years of illicit drug use. The correlations for school were negative, meaning the more years that an individual spent in school, the less likely he or she was to use an illicit drug. The significant predictors of frequency of illicit drug use, conditional on any use, were age, gender, and years-of-school. For instance, the frequency of stimulant and opiate use and number of years of regular illicit drug use (three or more times a week) varied as a function of age, gender, and years-of-school (as well as clinic status). Marijuana had a somewhat different profile in that age and gender were the only significant predictors of frequency of use. Thus, of the significant zero-order correlations in Table 2, years-of-school remained a robust predictor of drug use when other predictors were included in the analyses, whereas IQ, impulsivity, and psychiatric symptoms did not.

### Licit drugs

Table 4 shows that the regression results for licit drugs differed from those of the illicit drugs, particularly in the case of drinking bouts. The significant predictors of ever smoking (>100 cigarettes) were age, clinic stats, and years-of-school. The significant predictors of one or more drinking bouts (five or more drinks/occasion) were gender and impulsivity. The only significant predictor of number of cigarettes smoked among regular smokers was age. The significant predictors of the frequency of drinking bouts were gender and impulsivity. For the legal drugs, clinic status was not a predictor of frequency of use, and as with the illicit drugs IQ and psychiatric symptoms were no longer significant predictors of ever using a licit drug or the frequency of licit drug use, conditional on any use. However, impulsivity continued to predict drinking bouts in the multiple regression analyses.

### Age

Age was positively correlated with frequency of drug use for drug users. This could simply mean that older individuals had more time to use drugs. To test this inference, we repeated the analyses with rate of drug use as the dependent variable rather than frequency of drug use. If the relationship with age reflected nothing more than opportunity to use more, the correlation between age and rate of drug use would not be significant. The denominator for calculating rate of drug use was years of use (current age minus onset age). Age was not a significant predictor of rate of drug use.

### Years-of-school, impulsiveness, and IQ

**Figure 2:**
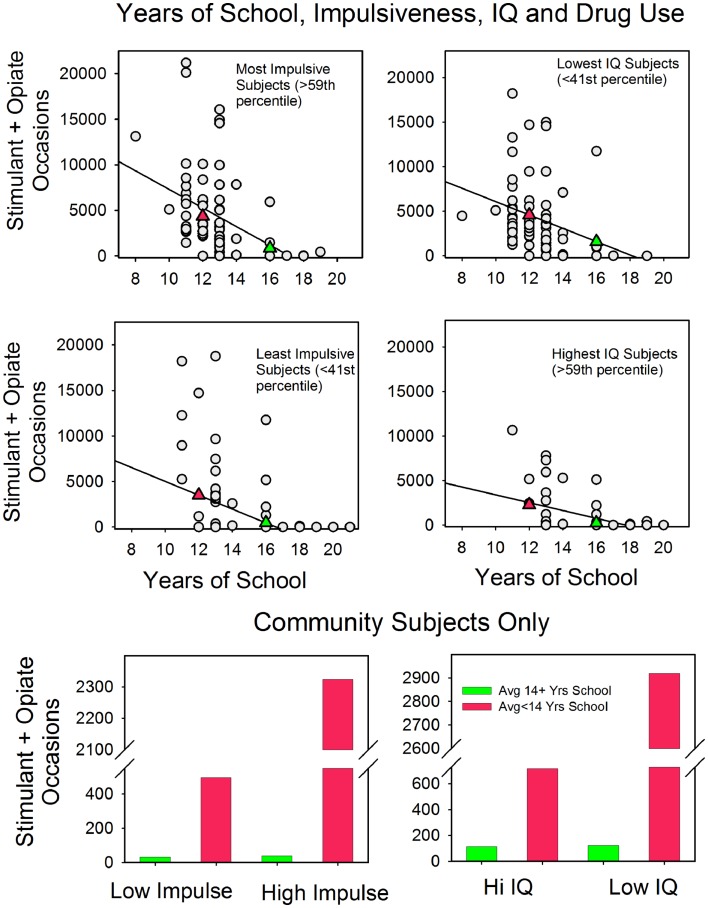
**The relationship between years-of-school and the frequency of stimulant and opiate use in the most and least at risk subjects (as measured by the Barratt Impulsiveness Scale and IQ)**. The top four panels show the findings from both clinic and community subjects. See text for details. The filled triangles indicate the averages for subjects with <14 years-of-school (red) and 14 or more years-of-school (green). The bottom two panels show these relations for just the community subject sample.

Figure 2 plots the relationship between years-of-school and stimulant and opiate use for the most and least impulsive subjects as measured by Barratt scores and for the least and most academically able subjects as measured by IQ scores (e.g., individuals who were “most impulsive” had a Barratt score above the 59th percentile, whereas individuals who were “least impulsive” had a Barratt score below the 41st percentile). Consider impulsiveness first (the left half of the graphs).

There was an inverse relationship between years-of-school and drug use for both the most and least impulsive subjects. Moreover, the relationship with school was strong enough to reverse the typical association between impulsiveness and drug use. For instance, a comparison of the top and middle panels shows that subjects who scored in the top two quintiles on the impulsivity questionnaire but who spent 14 years or more in school typically used stimulants and opiates less than did subjects who scored in the bottom two quintiles of the impulsivity scale but had spent <14 years in school (on average 848 and 3495 occasions, respectively). These two panels also show that for the more impulsive subjects there was about a fivefold increase in opiate and stimulant use for those with <14 years-of-school (4346 and 848 occasions), and the slopes of the fitted lines reveal that years-of-school had a slightly stronger association with differences in drug use for the more impulsive subjects.

To insure that years-of-school was not a proxy for clinic status, we tested the associations between school, impulsiveness, and drug use in a sample composed exclusively of community subjects. The bottom left panel shows the results. For subjects with <14 years-of-school, drug use differed markedly as a function of impulsiveness: 233 and 2048 occasions for low and high impulsiveness, respectively. However, community subjects who scored high on impulsivity but had 14 or more years-of-school used stimulants and opiates less frequently (233 occasions) than did community subjects who scored low on impulsivity but who went to school for fewer than 14 years (408 occasions). That is, for the community subjects, the typical link between impulsivity and drug use did not hold when years-of-school was included in the analyses.

The three panels on the right side of Figure 2 plot the relationship between years-of-school and drug use for high and low scorers on the IQ test. There was an inverse relationship between years-of-school and drug use for both groups, and as with impulsiveness, the relationship with school was strong enough to reverse the overall correlation between IQ and drug use. For instance, low IQ subjects with 14 or more years-of-school tended to use opiates and stimulants somewhat less than did high IQ subjects with <14 years-of-school (1593 and 2269 occasions on average, respectively). Also note that the slopes of the fitted lines imply that years-of-school had a slightly stronger relationship with drug use for the low IQ subjects, which parallels the results for differences in impulsiveness.

The bottom panel shows stimulant and opiate use as a function of IQ and school for community subjects only. The results parallel the impulsivity results. Community subjects who scored in the two lowest quintiles for IQ but attended school for 14 or more years used stimulants and opiates less frequently than did community subjects who scored in the top two IQ quintiles but who failed to go to school for 14 or more years (48 and 741 occasions, respectively).

## Discussion

The subjects in our study varied widely in terms of drug histories, educational history, and psychological characteristics, yet, the results were orderly. (1) In all but one case, the transformed frequencies of drug use approximated a normal distribution – and the probability plot for the one exception (marijuana) was quite similar to the others. (2) As in previous studies, the frequency of drug use was positively correlated with impulsivity and negatively correlated with cognition and years-of-school. (3) The magnitudes of the correlations varied as a function of type of drug, but the rank order of the correlations was the same for each drug. The correlations were stronger for verbal tests than for spatial tests and were stronger for working memory than for simple memory. (4) However, in the multivariate analyses, the relationship between cognition and frequency of drug use (conditional upon any use) was no longer significant. Similarly, the relationship between impulsivity and drug use did not remain significant, with the exception of drinking bouts. In contrast, the relationship between years-of-school and frequency of drug use remained significant for any stimulant and opiate use, any smoking, frequency of stimulant and opiate use, and years of regular illicit drug use. (5) In line with the multivariate dissociation of educational attainment and cognition, Figure 2 showed that high risk individuals who had been in school for 14 or more years used opiates and stimulants at lower levels than did low risk individuals with less schooling (where risk was defined by low IQ or high impulsivity). Similarly, among community subjects with 14 years or more of school, Figure 2 revealed that stimulant and opiate use was not associated with differences in impulsiveness or IQ. This is significant in that it suggests that with the exception of alcohol, time spent in school helps reduce drug use. However, before discussing these findings in more detail, there are several methodological issues to attend to. Are the results reliable, and are there reasons to expect that they apply beyond the individuals who served as the subjects in this report?

### Reliability of self-reported drug histories

Researchers have tested the reliability of self-reported drug use by comparing their informants’ words with physiological assays of drug use. The basic finding is that when the subjects appeared not to fear possible negative consequences for a candid account, self-reported levels of use approximated the metabolic estimates of levels of use. In contrast, when censure or worse was possible, participants under reported drug use (29–32). The present study approximated the conditions of the research projects that fostered reliable self-reported drug use. We guaranteed our informants anonymity, and we had no actual or apparent connection with the judicial system. In support of this point, and in line with previous findings, our verbal accounts of recent drug use indicated higher levels of recent use than did the metabolic tests. However, there is a second way to test the reliability of our self-report.

#### Experimental and self-report correlations

To determine the reliability of the subjects’ accounts of their drug histories, we can examine the correlations between the experimental session test results (e.g., working memory scores) and estimated frequencies of drug use based on self-report. If the reports are reliable then it is possible, although not necessary, that drug use frequency will correlate with the variables that the experimenter selected to study. However, if the self-reports are unreliable then such correlations could only appear by chance, and, accordingly, would be highly unlikely. For every drug years-of-school was the strongest correlate of frequency of use, for every drug verbal IQ was a stronger correlate of frequency of use than was spatial IQ, and for every drug working memory was a stronger correlate of frequency of use than was short-term memory. It is implausible that the subjects concocted drug histories that were so systematically related to their performance in the cognitive procedures. Put another way, the results are highly orderly, and it is much more plausible that this order reflects valid self-reports (and orderly correlations with cognition and psychological disposition) than accident or artifice.

### Generality of the results

We can compare our results with previous studies to evaluate the generality of the present findings. First, the zero-order correlations are consistent with scores of previous studies on the relations between educational attainment, impulsivity, and drug use, including large, national surveys that selected participants so as to match national demographic trends [e.g., Ref. (33)]. Similarly, the multiple regression analyses produced results that match previous findings. For instance, in a large prospective study of IQ and adult outcomes, Fergusson et al. (34), found that childhood IQ was correlated with educational attainment and a long list of dysfunctional adult behaviors. But then in the multivariate analyses with social covariates, IQ’s association with criminal activity, illicit drug use, and other dysfunctional activities shrank significantly. This is analogous to our results. We found strong zero-order correlations between IQ, years-of-school, and drug use, but IQ no longer predicted frequency of drug use when years-of-school was a covariate in the Double-Hurdle regression analyses. Thus, even though the present research recruited volunteers, the results match those of similar studies that used selection criteria that would necessarily reduce the biases that can accompany self-selected subjects.

### Alcohol

The pattern of correlations for bouts of heavy drinking differed from those of illicit drug use and smoking. Most notably, years-of-school did not predict heavy drinking, although impulsivity did. The most obvious explanation is that being in school limits access to illicit drugs but not to alcohol. This is consistent with recent research showing that dependence persists much longer for licit drugs than illicit drugs (35, 36). For instance, if years-of-school is negatively correlated with illicit drug use but not bouts of heavy drinking, then bouts of heavy drinking should persist longer, all else being equal.

### Interpretations of the findings

The multiple regression analyses show that years-of-school, clinical status, and gender remained significant predictors of the frequency of illicit drug use. Although each predictor is important to the understanding of drug use and addiction, we will focus largely on years-of-school. It was the strongest zero-order correlate of drug use, and it was typically the strongest zero-order correlate of the cognitive and dispositional correlates that were themselves significantly correlated with drug use.

#### The logical possibilities

There are three possible relationships between drug use and years-of-school: (1) drug use curtailed educational attainment, (2) educational attainment and drug use are not causally related but reflect one or more common factors, and (3) educational attainment and/or its correlates curtailed drug use. Each hypothesis has empirical support, and they are not mutually exclusive relations.

#### Did early drug use cut short time spent in school?

In a study with over a thousand subjects, Engberg and Morral (37) found that reducing drug use in young people increased school attendance. Their subjects were adolescents who had been admitted to drug treatment centers. This suggests that the correlations that we observed reflect in part or whole the negative influence of early drug use on staying in school. We examined this idea by evaluating the correlations between age of onset of drug use and years-of-school. If drugs curtailed school there should be a positive correlation between these two measures, i.e., younger age of onset and fewer years-of-school. For all subjects that had used an illicit drug one or more times, the correlation was in the expected direction, but small and not statistically significant (*r* = 0.09, *p* = 0.30). But perhaps the correlation would be stronger if the analysis was restricted to just those individuals who became regular illicit drug users (three times a week or more for at least a year)? The results were about the same (*r* = 0.07, *p* = 0.44). In similar analyses for drinking and smoking, those who started smoking at an earlier age tended to leave school earlier, but the correlation was weak (*r* = 0.17) and not statistically significant (*p* = 0.21). In contrast, the correlation between age of onset for binge drinking and years-of-school was negative, meaning there was a tendency for those who stayed in school longer to report more binge drinking episodes. However, as was the case for illicit drugs and smoking, the association was not statistically significant (*r* = −0.18, *p* = 0.07). Thus, for both licit and illicit drugs the relationship between age of onset of drug use and years-of-school was weak.

As a second check on the relationship between school and drug use, we compared the correlations between IQ and years-of-school for regular illicit drug users and not-regular illicit drug users. If drug use cut short school then it is reasonable to suppose that it also weakened the correlation between IQ and years-of-school (see, e.g., Table 2). The IQ and years-of-school correlation for regular drug users was *r* = 0.49 and for not-regular drug users it was *r* = 0.36. That is, IQ was a slightly better predictor of years-of-school in those who used illicit drugs more. Thus, for the subjects in this study, we did not find evidence that the onset of drug use cut short schooling.

That our results did not replicate those of Engberg and Morral (37) may reflect differences in the participants. Engberg and Morral’s subjects had already been admitted to drug treatment programs although they were still in their teen years. In contrast, the subjects in our study were not necessarily at risk for drug use as teenagers. Most – including the clinic subjects – had completed high school. Thus, it seems reasonable to suppose that drug use is much more likely to undermine education in youngsters who are already at risk for not finishing high school.

#### Is there a common underlying factor?

It is plausible that the factors that influence years-of-school also strongly influence years of drug use so that the two outcomes are different aspects of a single syndrome. In the limit, the “single syndrome” thesis predicts that there is no causal relationship between school and drug use. Cognition, impulsiveness, and psychiatric symptoms are reasonable common factors. However, years-of-school remained a unique predictor of drug use when these factors were included in the regression analyses. Thus, the limiting case that educational attainment has no causal tie to drug use (despite zero-order correlations) was not supported. It is easy, though, to imagine other common factors. For example, “conscientiousness” predicts performance in school [e.g., Ref. (38)] and drug use (39). Thus, in future studies on the role of education in drug use it would be of interest to measure the influence of conscientiousness and other personality traits.

#### Did years-of-school help limit drug use?

According to the regression analyses, years-of-school was a significant predictor of whether someone became a stimulant and opiate user or a smoker and of the frequency of stimulant and opiate use and years of regular use of any illicit drug. Although this is a cross-sectional study, the pattern of correlations is most simply understood in terms of the influence of years-of-school on drug use.

First, as has been emphasized, verbal cognition was a stronger predictor of drug use than was spatial cognition and this held for cigarettes and alcohol as well as for illegal drugs. There is no known pharmacological explanation for this result, and given the very disparate pharmacological properties of the drugs, it seems highly unlikely that one exists. However, the rank order of the correlations makes sense if years-of-school played a key role in limiting drug use. First, Table 2 shows that cognition, impulsivity, and psychiatric symptoms showed the same pattern of correlations with drug use as they did with years-of-school. Verbal cognition was more strongly correlated with both frequency of drug use and years-of-school than was spatial cognition. Similarly working memory scores were more strongly correlated with both frequency of drug use and years-of-school than was simple memory. The simplest interpretation is that the cognitive differences resulted in differences in educational attainment, which, in turn, led to differences in drug use. In support of the first point, researchers routinely find that verbal cognition is a better predictor of academic performance than spatial cognition [e.g., Ref. (40, 41)]. In support of the second point, longitudinal studies of at risk children repeatedly find that those children who do better in school, even in the elementary grades, have better adolescent and adult outcomes [e.g., Ref. (5, 42)]. That is, there are empirical precedents for the interpretation that school provides social and health benefits, such as less illicit drug use. (What is new is this report is that the correlation between educational attainment and drug use remained after controlling for IQ and impulsivity.)

### Limitations

We see several limitations: (1) the study relies on self-report, (2) the subjects were not selected randomly, (3) the analysis was correlational, and (4) we restricted the subjects to those who graduated high school or obtained a GED. We have discussed the first three, as it seemed essential to do so before considering the implications of the findings. The problem with the GED or high school graduation requirement is that it may have limited the range of values for years-of-school. Nevertheless, years-of-school was the best predictor of drug use, cognition, and psychiatric symptoms. Moreover, according to Figure 2 the critical number of years-of-school is 14 or more. This is close to the median value for the subjects in this study, so that the range of variation in years-of-school was appropriate for detecting the critical 14-year mark. These findings suggest that the most likely consequence of widening the educational criteria would be stronger evidence for the importance of education in drug use.

### Relevance to the understanding of addiction

#### Methodological relevance

Years-of-school was the strongest zero-order correlate of drug use and the most consistent predictor of illicit drug use in the multiple regression analyses. However, we would not have discovered this had we followed the more typical research methodology of studying just those drug users who were in treatment, as this would have restricted the range of variation in educational attainment. This raises the possibility that there may be other little studied variables that are also powerful predictors of drug use and/or variables, which predict both years-of-school and drug use. However, in order to investigate these questions researchers must include subjects who vary widely demographically, which will likely result in data sets with a large number of zeroes. We found that Cragg’s Double-Hurdle regression method offered a handy solution to this problem, and it should do the same for other researchers who recruit subjects from the community as well as from the clinic.

#### Conceptual relevance

Addiction is often referred to as a “chronic relapsing disease” [for discussion and history of this viewpoint, see Ref. (43)]. In line with this definition, the directors and spokespersons of the American federal addiction research institutes promote molecular, pharmacological accounts of excessive drug use [e.g., Ref. (44)]. They claim that drug use transforms voluntary drug experimenters into involuntary “addicts” who have lost the capacity to say “no” [e.g., Ref. (45)]. However, the results presented here support the idea that social processes, such as time spent in school, play an important role in drug use. In support of these findings, national surveys reveal that educational attainment is a potent predictor of who quits smoking cigarettes (46, 47) and who quits heavy drug use [e.g., Ref. (33)]. Moreover, according to the regression analyses what mattered for the subjects in this study was time in school, not its cognitive or dispositional correlates. Put more generally, although addiction researchers have emphasized individual differences in the likelihood that drug use leads to excessive use, it may turn out that historical and other social factors are at least if not more important. For example, cohort differences in prevalence are substantially larger for addiction than for other psychiatric disorders [Ref. (43), Figure 2.3].

#### Relevance for interventions

Figure 2 points to the potential practical significance of the results. For those subjects who were most at risk for stimulant and opiate use (as measured by IQ and impulsivity), years-of-school was associated with lower than expected levels of drug use. Indeed the high risk subjects with 14 years or more of school used opiates and stimulants less than did the low risk subjects who had <14 years-of-school. This is important. To our knowledge, there are no reliable programs for boosting IQ or curbing impulsivity. In contrast, efforts to increase schooling have been successful. For instance, in the United States over the last 10 years there has been about a 25% increase in the number of individuals aged 25 and older who completed college (48). This suggests that a plausible approach to excessive drug use is indirect: promote programs that increase post high school training. Also note that there is nothing in our results or those that we reviewed that say that such schooling has to be college oriented. In the present study, years-of-school included a wide range of programs, not just academic ones. In support of this point are the results from an interesting report on the relationships between time in the classroom, academic skills, and safe-sex. The researchers (49) measured years-of-school, reading ability, evidence of learning disabilities, and prudent sexual behavior among female prison inmates. Years-of-school predicted the likelihood of taking precautions against contracting HIV; language skills and learning disabilities did not. That is, how long the women went to school, not what they had learned in school, predicted healthy behaviors.

#### How might years in school influence drug use?

This last observation raises the issue of how might time spent in school have constrained drug use? Much has been written about this question [e.g., Ref. (50)]. Lleras-Muney (51), an economist, found that years-of-school was correlated with increased life span in the United States even after controlling for region of the country, occupation, access to medical care, and gender. She and a colleague, David Cutler and Lleras-Muney (52), speculate that “increasing levels of education lead to different thinking and decision-making patterns” that promote more prudent behavior. In a longitudinal study, Henry et al. (42) found that years-of-school markedly weakened the correlation between early childhood measures of impulsiveness and antisocial behavior as measured at age 21. Importantly, these effects were greatest for those who scored highest on the “Lack of Control” behavioral scale. The authors speculate that attending school strengthens ties to social institutions and values, and that this inhibits antisocial behavior, particularly in those who are most likely to be antisocial.

In addition to the possible cognitive and social benefits of time spent in school, Figure 2 suggests that school may function something like a physical barrier against frequent illicit drug use. The graphs show that school had a more pronounced effect on drug use for those who spent 14 or more years in the classroom. Put in terms of age, those with 14 years or more of school tended to be in a classroom at least part of the day during their late teens and early twenties. This is just the age at which heavy, illicit drug use typically starts [e.g., Ref. (53)]. Thus, participation in post high school education and professional programs may keep young adults “off the streets” at just the age when they are most likely to become frequent drug users. This not to say that school is a panacea. Individuals with secure careers become heavily involved with drugs (54), and most individuals who have <14 years-of-school do not become drug addicts. These two points do not undermine the role that school plays in promoting healthy behavior. Rather they simply show that school is not the only predictor of drug use.

## Conclusion

To our knowledge, this is the first study to simultaneously evaluate the correlations between impulsiveness, cognition, years-of-school, and drug use. The simplest interpretation of the results is that to a significant degree the cognitive and dispositional correlates of drug use (listed in Table 2) were in place prior to drug use, and that the negative correlations between years-of-school and illicit drug use and between years-of-school and smoking were due in some part to school itself, not its correlates. The practical significance of these findings is that programs that promote education and training, particularly in young adults, will pay dividends as measured by decreases in drug use. Moreover, the results suggest that such programs may be most useful for those most at risk. These are testable ideas and according to the results presented here, promising ideas.

## Conflict of Interest Statement

The authors declare that the research was conducted in the absence of any commercial or financial relationships that could be construed as a potential conflict of interest.
